# Co-Twin Control Models Indicate Genetic Confounding Between Education and Longitudinal Changes in Cognitive Aging

**DOI:** 10.1093/geroni/igaf122.1583

**Published:** 2025-12-31

**Authors:** Deborah Finkel, Malin Ericsson, Ida Karlsson, Miriam Mosing, Marianne Nygaard, Chandra Reynolds, Margaret Gatz, Brian Finch

**Affiliations:** University of Southern California, Los Angeles, California, United States; Karolinska Institutet, Solna, Stockholms Lan, Sweden; Karolinska Institutet, Solna, Stockholms Lan, Sweden; Karolinska Institutet, Solna, Stockholms Lan, Sweden; University of Southern Denmark, Odense M, Syddanmark, Denmark; University of Colorado Boulder, Boulder, Colorado, United States; University of Southern California, Los Angeles, California, United States; University of Southern California, Los Angeles, California, United States

## Abstract

Education is perhaps the most widely examined early life protective factor for cognitive decline. Potential mechanisms for the relationship include direct causation, indirect causation (education may reflect a more advantaged early life environment), or genetic confounding (genes associated with educational attainment may also impact cognitive aging). Twin studies offer a method for testing causal hypotheses by incorporating within and between twin pair differences in longitudinal latent growth curve models (LGCM) of cognitive aging on both level of functioning and rate of change with age. Nine longitudinal twin studies of aging from the IGEMS consortium (N = 23,269) included up to 27 years of follow-up on measures of cognitive function in the domains of memory, perceptual speed, verbal ability, and semantic fluency. Pair means (between family effect) and within pair differences (within family effect) were included as covariates of both intercept and slopes in age-based quadratic LGCM (centered at age 65). LGCM results were compared across full sample, monozygotic twins, and dizygotic twins. Results differed across cognitive tasks, but generally MZ within pair effects of education were strongly attenuated compared with DZ within pair effects, indicating some genetic confounding. Some sex differences in the results were identified, likely a result of the differential access to education among the birth cohorts represented in the sample (birthyears 1900-1948). Thus, results suggest shared genetic factors explain some of the association between education and longitudinal change in cognitive function. In other words, genes that impact educational achievement also influence cognitive aging.

